# Epidemiology of Corneal Neovascularization and Its Impact on Visual Acuity and Sensitivity: A 14-Year Retrospective Study

**DOI:** 10.3389/fmed.2021.733538

**Published:** 2021-10-14

**Authors:** Romina Mayra Lasagni Vitar, Giacinto Triolo, Philippe Fonteyne, Cecilia Acuti Martellucci, Lamberto Manzoli, Paolo Rama, Giulio Ferrari

**Affiliations:** ^1^Cornea and Ocular Surface Unit, Eye Repair Lab, Istituto di Ricovero e Cura a Carattere Scientifico (IRCCS) San Raffaele Scientific Institute, Milan, Italy; ^2^Ophthalmic Institute, ASST Fatebenefratelli-Sacco, Milan, Italy; ^3^Department of Medical Sciences, University of Ferrara, Ferrara, Italy

**Keywords:** corneal neovascularization, visual acuity, corneal sensitivity, retrospective study, epidemiology

## Abstract

**Purpose:** To quantify the severity and location of corneal neovascularization (cNV) and its impact on the visual acuity and corneal sensitivity in a cohort of the patients referred to a specialist cornea clinic and also to describe the etiology of cNV in the cohort.

**Methods:** We retrospectively evaluated the charts of 13,493 subjects referred to the San Raffaele Cornea Unit between January 2004 and December 2018 to search for cNV diagnosis. The corneal neovascularization severity was measured in the quadrants (range: 1–4) and location was defined as superficial, deep, or both. Best spectacle corrected visual acuity (BSCVA) was measured in logMar. We used the multiple regression analysis to identify the independent predictors of logMAR, after adjusting for age, gender, keratoconus, herpes keratitis, penetrating keratoplasty, trauma, and cataract surgery.

**Results:** Corneal neovascularization was diagnosed in 10.4% of the patients analyzed. The most prevalent etiology of cNV in our population was non-infectious corneal dystrophies/degenerations followed by herpes simplex virus infection. cNV affected OD, OS, or both eyes in 35.6, 40.2, and 24.2 of cases, respectively. Mean BSCVA (SD) was 0.59 (0.76), 0.74 (0.94), and 1.24 (1.08) in cNV one, two, and three or four of the quadrant groups. Superficial, deep, or mixed cNV occurred in 1,029, 348, and 205 eyes. Severe cNV (three or four of the quadrants) was a significant predictor of low visual acuity (*p* < 0.001) and reduced corneal sensitivity (*p* < 0.05). cNV location and its severity were associated (*p* < 0.05). In addition, corneal anesthesia was associated with lower BSCVA (*p* < 0.001).

**Conclusion:** Severe and deep cNV are associated with the reduced visual acuity and corneal sensitivity. Our data strongly support the relevance of appropriate follow-up as cNV is a major risk factor for graft rejection.

## Introduction

The incidence of corneal neovascularization (cNV) in the patients with ocular surface and its impact on visual acuity are not clear, although there is a general consensus that arresting cNV progression is beneficial (1).

A well-known immune-privileged site, the normal cornea is avascular (2, 3). A number of ocular diseases, generally associated with acute or persistent inflammation, results in an “angiogenic shift” and the development of cNV (4). The second cause of blindness worldwide, cNV, is a sight-threatening condition (5). Colby et al. reported a prevalence of 4.14% in a cohort of the patients visiting a general eye service (6).

The etiopathology of cNV varies across the world. In Western countries, herpes simplex keratitis is the most common infectious cause of cNV. In the US alone, 500,000 cases of ocular herpes simplex virus are reported every year (7). Contact lenses and specifically extended-wear soft contact lenses are the most frequent non-infectious cause of cNV in the US affecting 11–30% of the contact lens wearers (8). In addition, cNV invariably follows the corneal chemical burns with an incidence of 37,000 people/year in the US. Other diseases frequently associated with cNV include pterygium, Stevens–Johnson syndrome, Lyell syndrome, and limbal stem cell deficiency (9, 10). Of note, cNV is universally considered as a significant risk factor for corneal transplant rejection as the extent of cNV is directly related to the risk of rejection (11). This is a relevant clinical problem, since nearly 20% of the corneal buttons excised during corneal transplantation exhibit histological evidence of cNV (12).

Literature suggests that the diseases commonly associated with cNV affect visual acuity, the exact impact of cNV and its location on vision are unknown. At the same time, experimental evidence in the animal models of cNV shows impairment of the corneal nerves, but it is not clear if this phenomenon is replicated in human subjects.

In this study, we aimed to quantify the impact of cNV on the visual acuity and corneal sensitivity. Furthermore, we provided an estimate of cNV prevalence in the patients affected with ocular surface diseases.

## Materials and Methods

### Study Design

This retrospective analysis was conducted at the Cornea and Ocular Surface Unit of the San Raffaele Scientific Institute, Milan, Italy. The study was carried out in accordance with the guidelines established by the Declaration of Helsinki and the Institutional Review Board/Ethics Committee (Comitato EticoIstituto Scientifico Ospedale San Raffaele) approval was obtained.

All the data used in this study were extracted from our electronic medical record system (OCULI, Bedigital SrL, Verona, Italy), which includes all the patients evaluated at the Cornea and Ocular Surface Unit of the San Raffaele Scientific Institute, Milan, Italy, between January 1, 2004, and December 31, 2018. Specific database search included “history of systemic and/or ocular illness,” “best spectacle corrected visual acuity (BSCVA),” “slit-lamp biomicroscopy of the anterior segment” (including the extension of the cNVs in one to four quadrants), “corneal sensitivity” (quantified as present or absent), and “suspect/definitive diagnosis.” In this study, the following keyword combination was searched: “corneal neovascularization,” which retrieved all the patients affected with cNV in one or both eyes in at least one visit. If cNV was documented in both the eyes, they were both considered in the analysis. In total, the charts of 1,406 subjects were analyzed retrospectively.

### Clinical Parameters

Best spectacle corrected visual acuity was recorded in the Snellen equivalents and converted to logMAR scale. “Counting finger” (CF) and “hand motion” (HM) BSCVA were converted to 2.0 and 3.0 logMAR values, respectively; “light perception” (LP) and “no light perception” (NLP) BSCVA were not converted to any logMAR value (13) and were not considered in this study. Therefore, visual acuity data from the patients with BSCVA less than HM were not included in the analysis. Corneal sensitivity was assessed by using the cotton swab test.

Corneal neovascularization was defined as the presence of vessels in the cornea. The extension of cNV was quantified clinically as the number of the corneal quadrants showing neovessels (one, two, three, or four quadrants). cNV depth was evaluated clinically at the slit-lamp biomicroscopy. A cotton tip was used to evaluate corneal sensitivity, which was defined as normal, reduced, or absent (14).

The etiology of cNV was defined based on medical history, clinical suspicion, and/or confirmed microbiology testing. We identified two major groups: cNV associated with infectious keratitis or non-infectious cNV. The infectious keratitis group was further subclassified as follows: (i) viral keratitis: herpesvirus [herpes simplex virus (HSV) or varicella zoster virus (VZV)], adenovirus, and morbillivirus; (ii) bacterial keratitis: *Staphylococcus aureus, Pseudomonas aeruginosa, Streptococcus pneumoniae*, and *Chlamydia trachomatis*; (iii) *Acanthamoeba keratitis*; and (iv) fungal keratitis. In those cases where no definitive diagnosis was reached, either microbiological or clinical, the diagnosis was noted as “non-determined (ND).” The non-infectious keratitis group consisted of the following diagnosis: (i) trauma; (ii) allergic keratoconjunctivitis; (iii) chronic (i.e., more than 2 days per week) contact lens wear; (iv) dry eye and other immune-mediated disorders (rosacea, Sjögren's syndrome, and rheumatoid arthritis-associated ulcers); (v) ocular cicatricial pemphigoid and epidermolysis bullosa; (vi) Stevens–Johnson syndrome and Lyell syndrome; (vii) genetic/congenital/acquired dystrophies including keratoconus; (viii) chemical caustication; (ix) neurotrophic/neuroparalytic keratitis; (x) postocular surgery; and (xi) pterygium.

### Statistical Analysis

The Kolmogorov–Smirnov, D'Agostino–Pearson, and Shapiro–Wilk tests for normality failed for all considered variables and non-parametric testing was performed on the data. Multiple comparison tests of the Kruskal–Wallis and Dunn were applied to test the association between the visual acuity and cNV. In this case, statistical analysis was performed by using GraphPad Prism version 5.0 (GraphPad 22 Software, La Jolla, California, United States of America). Data are expressed as mean ± SD.

In addition, we evaluated the potential association between the corneal neovascularization severity and visual acuity by using multiple regression with logMAR as the dependent variable and adjusting for age, gender, keratoconus, herpetic keratitis, penetrating keratoplasty, trauma, and phacoemulsification + intraocular lens. Sensitivity and inflammation type (blepharitis, hyperemia, or both) were excluded from the models because of the very large number of missing values. When included, however, they did not affect the significant variables.

The validity of final regression models (left and right eye) was assessed as follows. The assumption of constant error variance was checked graphically, plotting Pearson residuals vs. fitted values, and, formally, using the Cook–Weisberg test for heteroskedasticity. Since Cook and Weisberg's test for heteroskedasticity was borderline significant, we used Huber/White robust SEs in the final models. High leverage observations were identified by calculating the Pearson, standardized, and studentized residuals, Cook's *D* influence, and the hat diagonal matrix. We found only 15 high-leverage observations excluding which we noted no substantial changes.

A multiple regression was also fit to evaluate the potential independent predictors of sensitivity. The same above criteria to set the final models (left and right eye) were used.

Statistical significance was defined as a two-sided *p* < 0.05 for all the analyses, which were performed by using STATA 13.1 (StataCorp LLC, College Station, Texas, United States of America).

## Results

Out of 13,493 patients who visited the Cornea and Ocular Surface Unit of the San Raffaele Scientific Institute, Milan, Italy between January 1, 2004, and December 31, 2018, a total of 1,406 (10.4; 95% CI 0.099–0.109) Caucasian patients (1,576 eyes) were diagnosed with cNV and included in the study. One thousand and sixty six patients showed monolateral cNV and 340 bilateral cNV. The mean age of the patient was 50.9 ± 20.2 years; males were 51.3% and females were 48.7%. LogMAR BSCVA was retrieved in 1,539 out of 1,576 eyes (37 eyes were not tested for BSCVA). Grading of cNV was performed in 1,456 eyes. The corneal sensitivity measurements were retrieved in 305 eyes (19.4%). The demographics of the patient are summarized in Table 1.

**Table 1:** Descriptive analysis of the sample of the patients with corneal neovascularization (cNV).

**Variables**	**Total sample (***n*** = 1,406)**	**Left eye**	**Right eye**
Male gender, %	51.3		
Mean age in years (SD)	50.9 (20.2)		
Age class, %			
<18 y	5.6		
18–64.99 y	65.7		
≥65 y	28.9		
cNV side, %			
- Left	35.6		
- Right	40.2		
- Both	24.2		
cNV sector, %	–	(*n* = 687)	(*n* = 769)
−1		32.6	33.9
−2		32.0	32.2
−3 or 4		35.4	33.8
cNV type, %	–	(*n* = 765)	(*n* = 819)
- Superficial		64.7	64.8
- Deep		22.0	21.9
- Mixed		13.3	13.3
Inflammation, %	(*n* = 620)		
- Blepharitis	31.8		
- Hyperemia	46.0		
- Both	22.3		
Visual acuity		(*n* = 741)	(*n* = 798)
MeanlogMAR (SD)	–	0.88 (0.98)	0.88 (0.96)
Sensitivity, %	–	(*n* = 149)	(*n* = 156)
- Absent		25.5	30.8
- Reduced		36.9	36.5
- Normal		37.6	32.7
Diseases*, %	(*n* = 1,254)		
- HSV keratitis	22.6		
- Penetrating keratoplasty	17.4		
- Keratoconus	14.8		
- Trauma	11.5		
- Keratitis, nc	9.2		
- Phacoemulsification + intraocular lens**	8.6		
- Failure**	7.9		
- Contact lens	5.3		
- Bacterial keratitis	3.1		
- Amebic keratitis	3.0		
- Leucoma, nc	3.0		
- Keratopia	2.8		
- Lamellar keratoplasty	2.7		
- Dry eye	2.7		
- Keratoconjuntivitis	2.5		
- Ulcer	2.2		
- Pemphigoid	1.9		
- Pterygium	1.6		
- Neurotrophic keratitis	1.6		
- Fuchs syndrome	1.3		
- Fungal keratitis	1.0		
- Salzmann syndrome	1.0		
- Autoimmune keratitis**	0.9		
- Steven Johnson syndrome	0.8		
- Sjorgen syndrome	0.8		
- Trans-pars plana vitrectomy	0.7		
- Glaucoma	0.6		
- Aniridia	0.6		
- Lyell syndrome	0.6		
- Viral keratitis excluding HSV	0.6		
- Terrien syndrome	0.5		
- Graft vs. host disease	0.3		
- Keratoglobus	0.2		
- Lasik	0.2		
- Coffin Siris syndrome	0.1		
- Bowen syndrome	0.1		
- Vogt syndrome	0.1		
- Goldenhar syndrome	0.1		
- Francois syndrome	0.1		

* *Patients may have been diagnosed with more than one disease*.

** *Total sample = 1,406*.

*nc, not classified*.

### Etiology of Corneal Neovascularization

**Figure 1:**
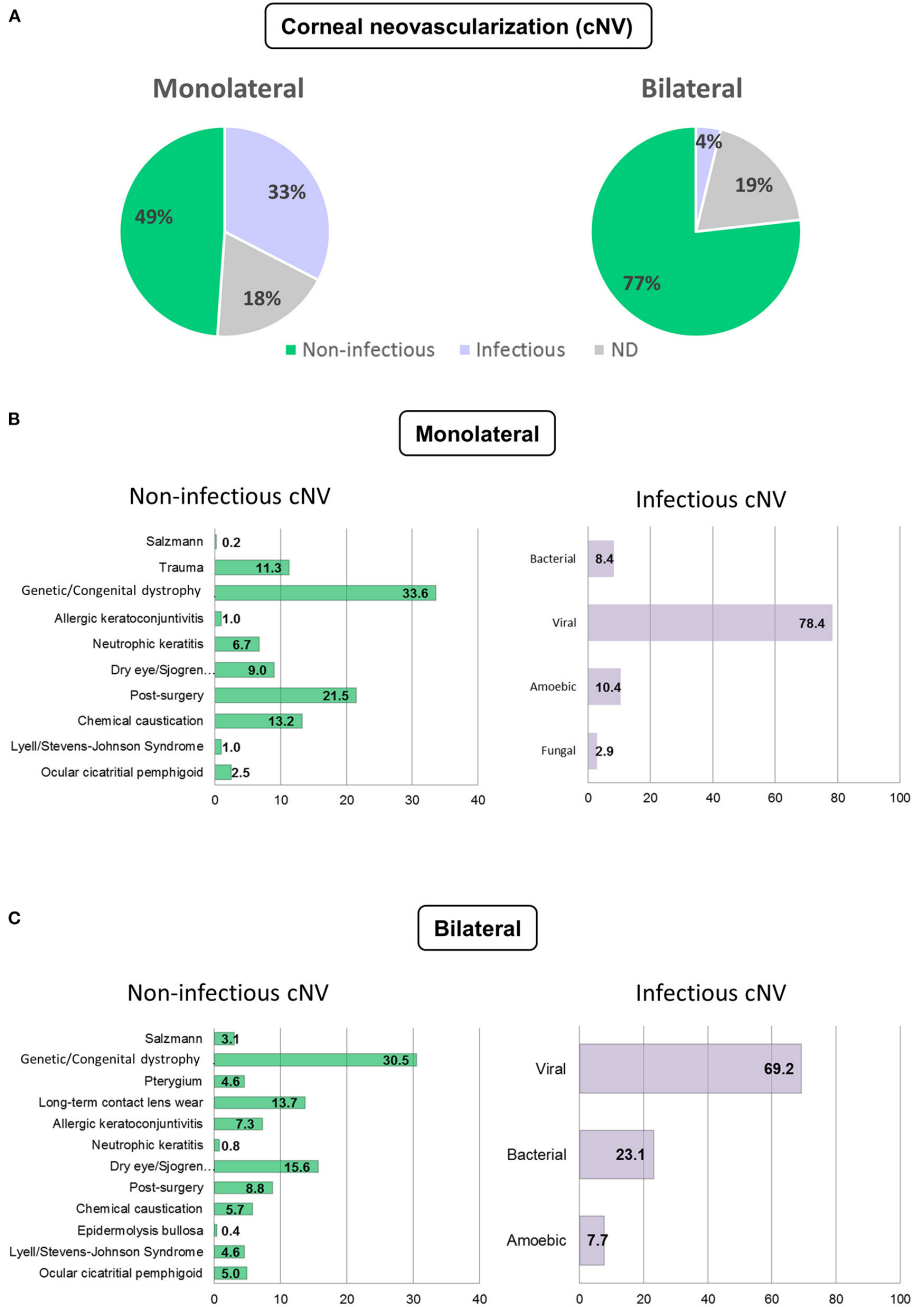
Etiology of corneal neovascularization (cNV). Etiology of monolateral and bilateral cNV that are classified as infectious, non-infectious, and non-determined (ND) **(A)** Distribution of the patients with infectious and non-infectious keratitis in monolateral **(B)** and bilateral **(C)** cNV.

The etiology of cNV was most frequently non-infectious in both the monolateral (48.9%) and bilateral (76.8%) cases (Figure 1A).

In case of monolateral cNV, the etiology was infectious keratitis in 32.6% of the cases and undetermined in 18.5% of the cases. Viral keratitis (69.3%) was followed by bacterial (23.1%) and amebic (7.7%). Among non-infectious keratitis, the most prevalent diagnoses were genetic/congenital dystrophies, including aniridia, Fuchs and Terrien dystrophies, keratoconus, and others (33.6%). This was followed by cNV occurring after ocular surgery (21.5%), chemical caustication (13.2%), trauma (11.3%), cNV associated with dry eye, and other autoimmune diseases (9%). Ocular surgeries included: phacoemulsification and extracapsular cataract extraction (ECCE) with intraocular lens (IOL) implant, trans pars plana vitrectomy (TPPV), refractive surgery, pterygium removal, lamellar keratoplasty (LK), and penetrating keratoplasty (PK). Other causes of less prevalent non-infectious cNV were: neurotrophic keratitis (6.7%), ocular cicatricial pemphigoid (2.5%), Lyell and Stevens–Johnson syndromes (1%), and allergic keratoconjunctivitis (1%). These data are summarized in Figure 1B.

The etiology for bilateral cNV was non-infectious in 76.8% of the cases, infectious in 3.8% of the cases, and undetermined in 19.4% of the cases. Viral keratitis was the most prevalent infectious cause (69.3%) followed by bacterial (23.1%) and amebic (7.7%). Among non-infectious etiologies, genetic/congenital dystrophies were prevalent (30.5%) followed by dry eye, isolated or associated with Sjögren's syndrome, rosacea, or rheumatoid arthritis (15.6%). Long-term contact lens wear accounted for 13.7% of the cases, ocular surgeries for 8.8% of the cases, allergic conjunctivitis for 7.3% of the cases, bilateral chemical burns for 5.7% of the cases, while ocular cicatricial pemphigoid and Stevens–Johnson syndrome accounted for 5 and 4.6% of the cases, respectively. Other less frequent causes of non-infectious cNV are listed in Figure 1C. Categorization of cNV of the most frequent diseases is provided in Supplementary Figure 1.

### Visual Acuity and Corneal Neovascularization

**Figure 2:**
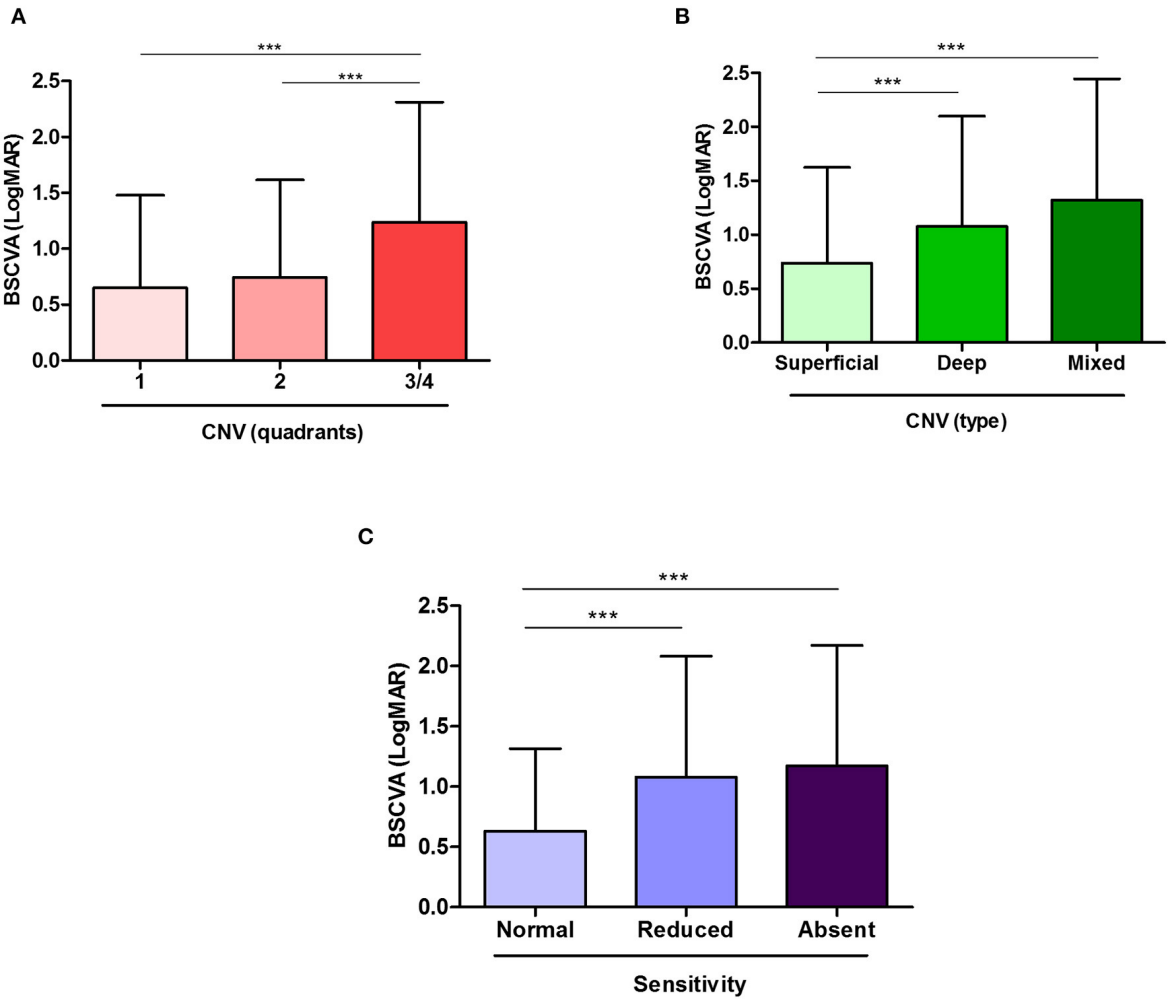
Visual acuity is affected by the extent of corneal neovascularization (cNV) and the decrease in corneal sensitivity. Worse best spectacle corrected visual acuity (BSCVA), observed as an increase in logMAR, is associated with increasing cNV extent **(A)** and location **(B)**. In addition, the patients with reduced or absent corneal sensitivity showed worse BSCVA **(C)**. Results are expressed as mean ± SD. Statistical analysis was performed by using multiple comparison tests of the Kruskal–Wallis and Dunn's. ***p < 0.001.

Out of the 1,539 eyes in which visual acuity was measured, cNV extension quantification was available for 1,229 eyes, which were used for this analysis. Eyes affected with one quadrant of cNV were significantly more likely to have better BSCVA than eyes presenting with three-fourths of the quadrants (*p* < 0.001). Similarly, eyes with two quadrants had significantly better BSCVA than eyes presenting with three-fourths of the quadrants of cNV (*p* < 0.001) (Figure 2A). Specifically, the media LogMAR was 0.65 ± 0.83, 0.74 ± 0.87, and 1.24 ± 1.08 in cNV one, two, and three-fourths of the quadrants, respectively. Moreover, severe cNV (three-fourths of the quadrants) was a significant predictor of low visual acuity (*p* < 0.001) (Table 2). In addition, the age of the patients with cNV was also negatively correlated with visual acuity (*p* < 0.001), while gender was not determinant (Table 2). Finally, superficial, deep, or mixed cNV occurred in 856, 281, and 190 eyes, respectively. We observed that, despite the amount of quadrants affected, most cNV was superficial (*p* < 0.05) (Table 3). In addition, eyes affected with superficial cNV presented significantly better BSCVA compared to eyes with deep and mixed cNV suggesting that lower visual acuity was associated with the extension and depth of cNV (*p* < 0.005) (Figure 2B and Table 4).

**Table 2:** Multivariate analysis predicting visual acuity (logMAR).

**Variables**	**Left eye**		**Right eye**	
	**Regression coefficient (95% CI)**	***p*** *	**Regression coefficient (95% CI)**	***p*** *
Age, 1-year increase	0.011 (0.006; 0.015)	<0.001	0.011 (0.007; 0.014)	<0.001
Male gender	0.053 (−0.113; 0.218)	0.5	−0.009 (−0.155; 0.137)	0.9
cNV, sector 1	0 (Ref. cat.)	–	0 (Ref. cat.)	–
cNV, sector 2	0.114 (−0.068; 0.296)	0.2	0.054 (−0.106; 0.214)	0.5
cNV, sector 3-4	0.542 (0.348; 0.735)	<0.001	0.512 (0.326; 0.698)	<0.001

* *Multiple regression with robust SEs. Number of observations: 493 (left eye) or 576 (right eye); R-squared: 0.206 (left eye) or 0.204 (right eye). Inflammation and sensitivity were excluded from the models because of the very large number of missing values. When included, however, they did not affect significant variables. An increase in logMAR regression coefficient corresponds to a decrease in visual acuity*.

**Table 3:** Association between corneal neovascularization (cNV) severity and the selected variables: univariate analyses.

**Variables**	**Left eye**				**Right eye**			
	**cNV 1**	**cNV 2**	**cNV 3-4**	***p*** *	**cNV 1**	**cNV 2**	**cNV 3-4**	***p*** *
	**(***n*** = 224)**	**(***n*** = 220)**	**(***n*** = 243)**		**(***n*** = 261)**	**(***n*** = 248)**	**(***n*** = 260)**	
Male gender, %	45.1	49.6	50.6	0.5	51.3	52.0	50.4	0.9
Mean age in years (SD)	47.6 (19.8)	50.9 (18.7)	52.5 (20.2)	0.03	47.4 (20.8)	49.8 (21.0)	52.4 (20.0)	0.03
Bilateral CNV, %	26.3	38.6	51.0	<0.001	23.0	35.1	46.9	<0.001
cNV sector on the other eye, %								
−1	69.0	14.6	5.8	<0.001	67.8	14.8	5.0	<0.001
−2	20.7	69.5	9.9		20.3	70.4	10.7	
−3/4	10.3	15.6	84.3		11.9	14.8	84.3	
cNV type, %	(*n* = 201)	(*n* = 200)	(*n* = 225)	0.05	(*n* = 230)	(*n* = 219)	(*n* = 242)	0.001
- Superficial	59.2	68.0	64.4		64.4	67.6	62.0	
- Deep	27.9	18.5	17.8		27.8	16.0	20.2	
- Mixed	12.9	13.5	17.8		7.8	16.4	17.8	
Visual acuity	(*n* = 201)	(*n* = 189)	(*n* = 190)	<0.001	(*n* = 232)	(*n* = 204)	(*n* = 213)	<0.001
MeanlogMAR (SD)	0.59 (0.76)	0.74 (0.94)	1.24 (1.08)		0.71 (0.88)	0.75 (0.80)	1.23 (1.07)	
Sensitivity, %	(*n* = 39)	(*n* = 33)	(*n* = 40)	0.04	(*n* = 60)	(*n* = 43)	(*n* = 35)	0.6
- Absent	20.5	21.2	42.5		25.0	34.9	31.4	
- Reduced	38.5	57.6	30.0		35.0	39.5	40.0	
- Normal	41.0	21.2	27.5		40.0	25.6	28.6	
Most frequent diseases, %	(*n* = 196)	(*n* = 180)	(*n* = 214)		(*n* = 232)	(*n* = 220)	(*n* = 230)	
- HSV keratitis	27.0	21.7	12.1	0.001	26.7	22.3	13.9	0.003
- Keratoconus	16.8	15.6	12.1	0.4	16.8	15.9	12.2	0.4
- Penetrating keratoplasty	13.8	16.7	19.2	0.3	15.5	16.4	14.4	0.8
- Trauma	6.1	7.2	12.6	0.04	8.2	12.7	17.8	0.008
- Phacoemulsification + intraocular lens	5.8	5.9	9.5	0.2	6.1	8.1	8.1	0.6

* *Kruskal–Wallis test for continuous variables, chi-squared test for categorical ones*.

**Table 4:** Association between corneal neovascularization (cNV) type and the most frequent diagnoses: univariate analyses.

**cNV type**	**Left eye**				**Right eye**			
**Variables**	**Superficial**	**Deep**	**Mixed**	***p*** *	**Superficial**	**Deep**	**Mixed**	***p*** *
	**(***n*** = 495)**	**(***n*** = 168)**	**(***n*** = 102)**		**(***n*** = 531)**	**(***n*** = 179)**	**(***n*** = 109)**	
Male gender, %	48.9	49.4	44.1	0.6	50.1	52.5	52.3	0.8
Mean age in years (SD)	49.5 (19.9)	50.7 (18.9)	52.6 (19.1)	0.3	48.5 (21.3)	49.6 (19.9)	57.6 (18.2)	<0.001
Visual acuity	(*n* = 430)	(*n* = 142)	(*n* = 81)		(*n* = 455)	(*n* = 141)	(*n* = 95)	
Mean logMAR (SD)	0.75 (0.91)	1.03 (1.00)	1.23 (1.10)	<0.005	0.73 (0.86)	1.12 (1.05)	1.40 (1.14)	<0.005
Most frequent diseases, %	(*n* = 420)	(*n* = 157)	(*n* = 91)		(*n* = 462)	(*n* = 163)	(*n* = 101)	
- HSV keratitis	13.1	25.5	30.8	<0.005	12.6	31.3	31.7	<0.005
- Penetrating keratoplasty	16.2	14.0	14.3	0.8	12.3	17.2	15.8	0.3
- Keratoconus	17.9	12.1	5.5	0.006	17.8	12.9	5.9	0.007
- Trauma	7.9	7.6	15.4	0.06	12.8	8.6	16.8	0.13
- Phacoemulsification + intraocular lens	7.1	10.2	8.8	0.4	7.9	8.4	9.2	0.9

* *Kruskal–Wallis test for continuous variables, chi-squared test for categorical ones*.

### Corneal Anesthesia and BSCVA

Out of 1,229 eyes where cNV was quantified, 253 eyes were also tested for corneal sensitivity, which were used for this analysis. BSCVA (LogMAR) was significantly lower (i.e., better vision) in eyes that presented normal corneal sensitivity (0.63 ± 0.68, *N* = 82) compared to the ones that showed reduced (1.08 ± 1.00, *N* = 100) or completely absent (1.17 ± 1.00, *N* = 71) (*p* < 0.001) sensitivity (Figure 2C). Moreover, we found that in the patients with cNV, lower visual acuity is a predictor of reduced corneal sensitivity (*p* < 0.05) (Supplementary Table 1).

### Corneal Neovascularization and Sensitivity

Out of 305 eyes where corneal sensitivity was measured, cNV was quantified in 203 eyes, which was included for the analysis. Table 3 shows the loss of corneal sensitivity that was associated with the extent of cNV (*p* < 0.05). In other words, the majority of the patients affected with cNV one quadrant presented normal sensitivity, while most of the patients with severe cNV (third-fourths of the quadrants) displayed anesthesia (*p* < 0.05). Therefore, cNV extent resulted in a significant predictor of lower corneal sensitivity (*p* < 0.05) (Supplementary Table 1).

## Discussion

Corneal neovascularization is the second cause of blindness worldwide (5) and an area of significant medical need. Current treatments include topical application of corticosteroids or non-steroidal anti-inflammatory agents, which can be associated with serious side effects such as ocular hypertension, posterior cataract induction, delayed healing, or corneal melting, respectively (4). Recently, vascular endothelial growth factor (VEGF) inhibitors such as ranibizumab (Lucentis; Genentech), and bevacizumab (Avastin; Genentech) have been proposed as a treatment for cNV with encouraging results (15, 16).

Inhibition of cNV has been considered clinically beneficial, although weak evidence supports the efficacy of the anti-cNV treatments in ameliorating vision (17). In any case, the real impact of cNV, and its extension, on visual acuity remains largely unknown. For this reason, we aimed to quantify the impact of cNV on visual acuity in a large population. We show that BSCVA is significantly and negatively affected by cNV in our cohort of 1,406 patients. To the best of our knowledge, only one prior report has assessed the impact of cNV on visual acuity and has found it reduced in 12% of patients affected with cNV (6), which is very similar to the prevalence reported in 10.4% of the patients. However, the limited number of the patients considered in that study (35 subjects out of 845 subjects) and the different population (general ophthalmology as opposed to cornea clinic) and the absence of cNV grading make it difficult to compare the two studies.

We also found that BSCVA is progressively reduced with an increasing extension of cNV. We would like to clarify that this does not mean that reducing the extent of cNV (therapeutically) may necessarily result in the improvement of BSCVA. In fact, vessel leaking of calcium or blood could irreversibly impair corneal clarity, and, hence, vision. Our study, however, suggests the importance of modulating cNV in its earlier stages as its progression to involve the entire cornea is indeed associated with worse vision. Additionally, we observed a significant correlation between cNV and loss of corneal sensitivity in line with prior reports in the animal models (18). The link underlying corneal nerve loss/dysfunction and cNV is not clear. It is known that corneal infections (19) or dry eye (20) is associated with altered nerve morphology and corneal sensitivity. One could hypothesize that neovessels induce corneal edema and, hence, cause peripheral nerve degeneration. Additionally, it is possible that normal nerves secrete anti-angiogenic factors such as pigment epithelium-derived factor (18), which are lost after their degeneration. Acute damage of corneal nerves and subsequent release of substance P could also promote cNV (21). Finally, it is possible that massive leukocyte infiltration, as it occurs during corneal inflammation, induces cNV and nerve disruption. Indeed, it has been reported that reduction of corneal nerve density is associated with an increased leukocyte infiltration in the conditions commonly associated with cNV (22).

Regardless of the mechanism(s) involved, this study suggests that corneas affected with cNV should be routinely checked for abnormal sensitivity to rule out concomitant neurotrophic disease.

Finally, we found that eyes with corneal anesthesia had worse BSCVA as opposed to eyes with normal corneal sensitivity. This suggests that vision reduction in patients with cNV could have multiple causes beyond the obvious effect of opacity induced by vessel growth into the cornea. In fact, impairment and/or loss of corneal sensitivity is associated with tear film alteration, punctate keratopathy, or ulcers, which can all reduce visual acuity. The finding that cNV patients exhibited altered nerve function should be taken into account, since anti-VEGF treatments, which have been proposed for cNV, are neurotoxic (23). Interestingly, VEGF neutralization with bevacizumab resulted in downregulation of nerve growth factor and delayed wound healing (24).

In summary, the most prevalent etiology of cNV in our population was non-infectious corneal dystrophies/degenerations. This group included, not surprisingly, the patients affected with chronic corneal edema or aniridia. The finding that the patients with keratoconus were also affected could be associated with extensive contact lens use in this group. On the other hand, infectious keratitis was the second cause of monolateral cNV and the third cause of bilateral cNV. This is in line with prior literature, which confirms a lower prevalence of infectious keratitis in developed countries (25). Among infections, herpes keratitis was the most prevalent, which consolidates prior reports (7). Of note, the most common diagnosis among the patients affected with most severe (third-fourths of the quadrants) cNV were penetrating keratoplasty and trauma (16.7 and 15.3%, respectively) followed by keratoconus (12.2%) (Supplementary Material).

We acknowledge that this study did not consider the central extension of cNV, as it was not possible to retrieve such information in this large cohort of patients. However, this study shows that higher cNV density (and, specifically, deep and extensive cNV) is associated with a lower vision that underlines the relevance of cNV-associated disorders as major drivers of visual impairment.

## Data Availability Statement

The raw data supporting the conclusions of this article will be made available by the authors, without undue reservation.

## Ethics Statement

The studies involving human participants were reviewed and approved by mds. Written informed consent from the participants' legal guardian/next of kin was not required to participate in this study in accordance with the national legislation and the institutional requirements.

## Author Contributions

GF and PR contributed to conception and design of the study. RL, GT, and PF organized the database. LM and CA performed the statistical analysis. RL, PF, and GF analyzed the data. RL and GF wrote the manuscript. All authors contributed to manuscript revision, read, and approved the submitted version.

## Funding

This work was supported by the institutional funding.

## Conflict of Interest

The authors declare that the research was conducted in the absence of any commercial or financial relationships that could be construed as a potential conflict of interest.

## Publisher's Note

All claims expressed in this article are solely those of the authors and do not necessarily represent those of their affiliated organizations, or those of the publisher, the editors and the reviewers. Any product that may be evaluated in this article, or claim that may be made by its manufacturer, is not guaranteed or endorsed by the publisher.
