# Supplementary material for: Epidemiology of Corneal Neovascularization and Its Impact on Visual Acuity and Sensitivity: A 14-Year Retrospective Study
Source: Front Med (Lausanne). 2021 Oct 14;8:733538. doi: 10.3389/fmed.2021.733538 (PMC8551447; doi:10.3389/fmed.2021.733538)
Supplement: Supplementary file 1 [file Data_Sheet_1.docx]

**Supplementary material**

**Supplementary Table 1**. Multivariate analysis predicting sensitivity *.

|  | **Left eye** |  |  | **Right eye** |  |
| --- | --- | --- | --- | --- | --- |
| Variables | Regression coefficient  (95% CI) | p** |  | Regression coefficient  (95% CI) | p** |
|  |  |  |  |  |  |
| cNV, sector 1 | 0 (Ref. cat.) | -- |  | 0 (Ref. cat.) | -- |
| cNV, sector 2 | -0.199 (-0.614; 0.216) | 0.3 |  | -0.452 (-0.808; -0.096) | 0.013 |
| cNV, sector 3/4 | -0.445 (-0.839; -0.051) | 0.027 |  | -0.083 (-0.462; 0.295) | 0.7 |
|  |  |  |  |  |  |
| logMAR, 1-unit increase | -0.210 (-0.400; -0.020) | 0.031 |  | -0.218 (-0.395; -0.042) | 0.016 |
|  |  |  |  |  |  |

* Categorized as 0=absent, 1=reduced, 2=normal; An increase in sensitivity regression coefficient corresponds to an increase in the likelihood of normal sensitivity.

** Multiple regression adjusting for age, gender, keratoconus, herpetic keratitis, penetrating keratoplasty, trauma, and Phacoemulsification + intraocular lens. Since none of these covariates were significant (p>0.2), they have not been shown to avoid redundancy. Number of observations of the model: 91 (left eye) or 112 (right eye); R-squared: 0.158 (left eye) or 0.132 (right eye). Inflammation was excluded from the models because of the very large number of missing values. When included, however, it did not affect significant variables..

**Supplementary Figure 1**

**
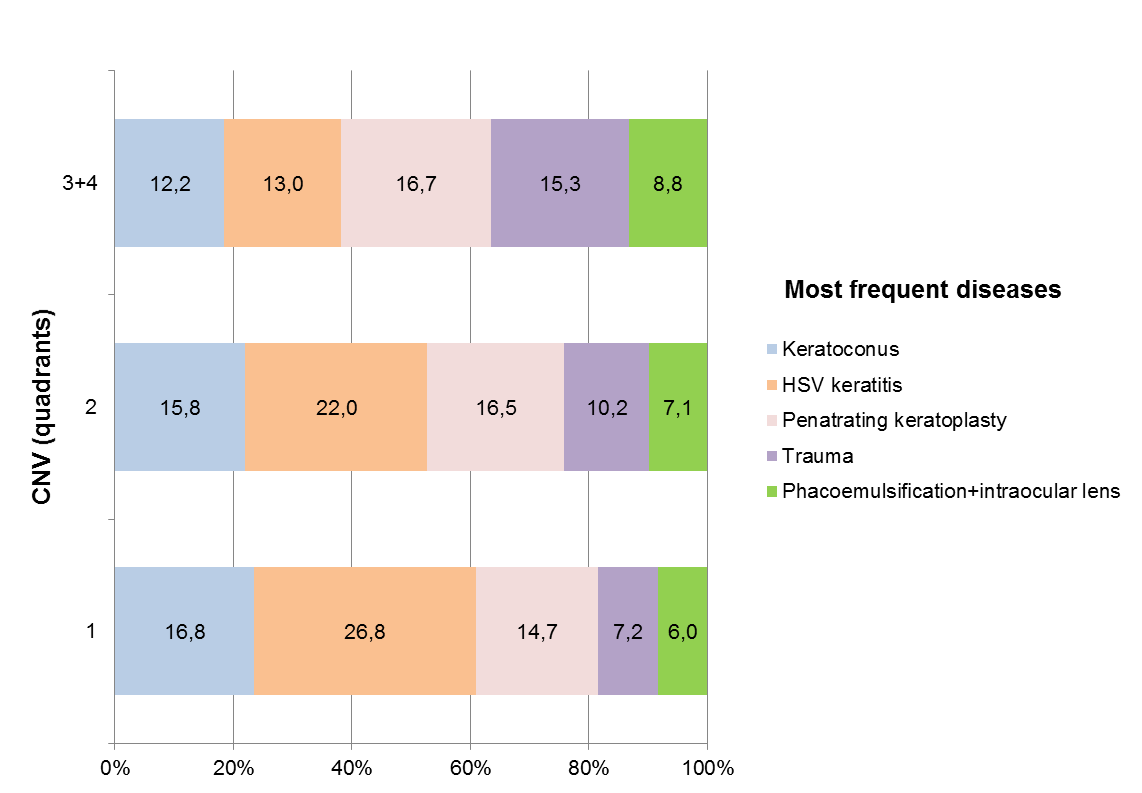
**

**A**

**cNV (quadrants)**


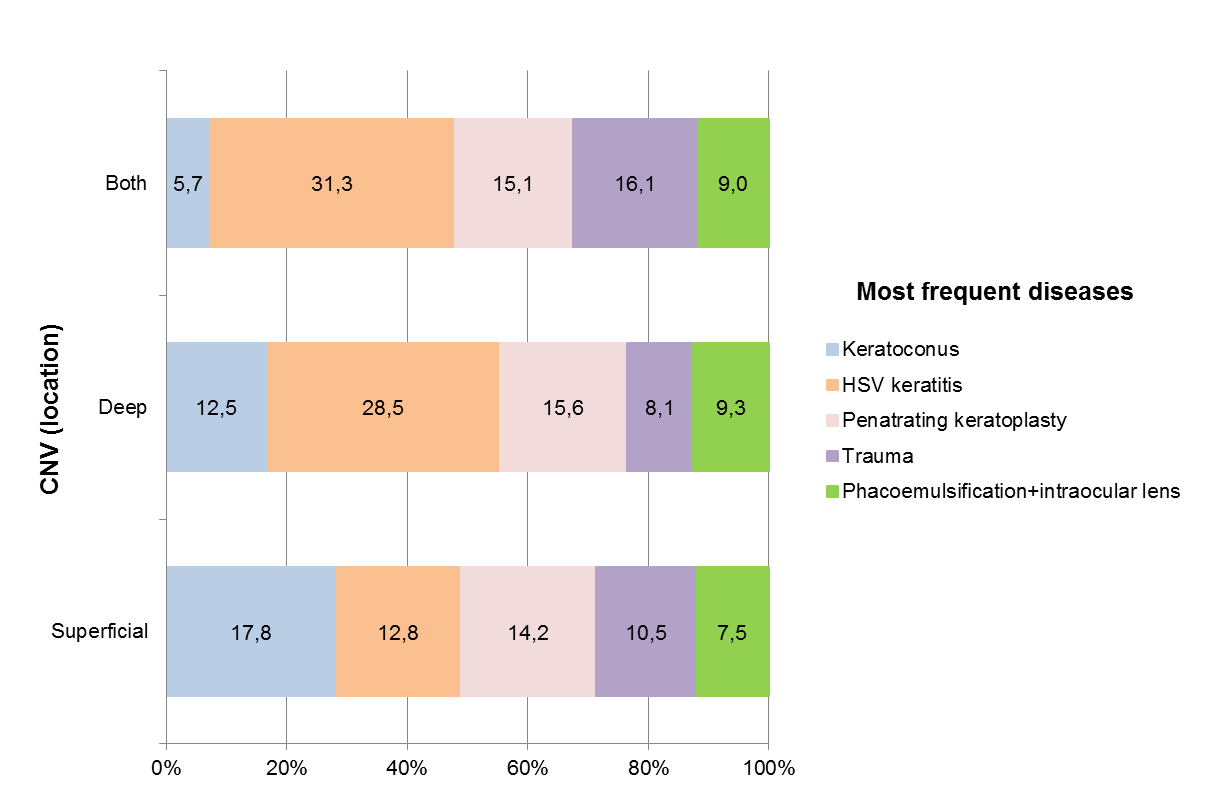


**B**

**cNV (location)**

**Supplementary Figure 1.** Corneal neovascularization (cNV) extent (A) and location (B) of patients with most frequent diseases.
